# Social support in a large general population sample over the course of six years

**DOI:** 10.1038/s41598-025-90703-y

**Published:** 2025-02-21

**Authors:** Andreas Hinz, Peter Esser, Diana Richter, Antje Schönfelder, Heide Glaesmer, Antje Lehmann-Laue, Svenja Heyne, Katja Leuteritz, Annekathrin Sender, Franziska Springer, Heide Götze, Anja Mehnert-Theuerkauf

**Affiliations:** Department of Medical Psychology and Medical Sociology, University of Leipzig, Philipp-Rosenthal-Str. 55, 04103 Leipzig, Germany

**Keywords:** Social support, Sociodemographic correlates, Longitudinal study, Social isolation, General population, Public health, Epidemiology

## Abstract

Social support is an important resource that is assumed to buffer the effect of stressful events on health. The aims of this study were to test psychometric properties of the ENRICHD Social Support Instrument (ESSI), to investigate the impact of several sociodemographic and behavioral variables on social support, and to analyze changes in social support over a 6-year period. A sample of 9,681 people from the general population was examined at baseline, 4,987 of whom were surveyed at a follow-up examination six years later using the ESSI and several other questionnaires. The psychometric properties of the ESSI were good (Cronbach’s α = 0.91) and measurement invariance across gender and age could be established. High socioeconomic status, sharing a household with others, and employment resulted in high levels of social support. Tobacco smokers and alcohol drinkers reported having less social support than nonsmokers and non-drinkers. During the 6-year period, the mean level of social support remained nearly unchanged (*d* = 0.01). The data provide a framework for the interpretation and comparison of social support with other clinical and nonclinical populations. Public health initiatives should aim to prevent social isolation to improve public health.

Social support is a psychosocial resource that is accessible in the context of interpersonal contacts^1,2^. Though there is no consensus on the precise conceptualization, most researchers think of social support as being composed of two components: structural support, which regards the size of the social network and the frequency of contacts, and functional support, which refers to the support a person thinks they can expect to receive from others if they need it^3^. Functional support is seen as being composed of two components, instrumental support and emotional support^4^. From an overarching conceptual perspective, social support can be considered one of those resources such as sense of coherence^5^, self-efficacy^6^, resilience^7^, and habitual optimism^8^ that buffer the impact of stressful events on health and quality of life.

Social support is relevant from a public health perspective because it is associated with self-rated health^9^, quality of life^10,11^, well-being^12^, low degrees of depression and anxiety^3,13^, gambling behavior^14^, health care adherence^15^, physical activity^16^, low prevalence of mental disorders^17^, low risk for coronary heart disease^18,19^and dementia^20^, and even lower all-cause mortality^21,22^.

Frequently used instruments for measuring social support are the Enhancing Recovery in Coronary Heart Disease (ENRICHD) Social Support Instrument (ESSI)^23^, the Oslo Social Support Scale in its 12-item long form or the 3-item short form^24^, the Duke Social Support Index^25^, and the Lubben Social Network Scale^26^. In addition, many quality of life questionnaires include a social support or social functioning subscale. The ESSI is a suitable five-item instrument for effectively measuring social support. In the terminology mentioned above, the ESSI captures the functional component of social support, including components of instrumental and emotional social support. Its brevity, general applicability, psychometric quality, and the presence of norm scores distinguish it as a suitable instrument for assessing social support in epidemiological studies. The reliability of the instrument proved to be good in several studies^27–29^, however, its measurement invariance across gender and age has yet to be tested.

Several studies have been performed to analyze the effect of gender, age, and other sociodemographic variables on levels of social support. Social support proved to be high when the respondents were married, employed, and had a high income, high level of education, and high socioeconomic level^28,30^. Concerning gender and age, the results were inconsistent. A general population study performed with the ESSI found higher scores for men than for women (effect sizes *d*= 0.15^29^); another general population study with the OSSS-3 found slightly higher scores for women (*d*= 0.03)^24^; and, using the Lubben scale, men showed higher frequencies of social isolation than women with an odds ratio of 1.49^30^. Regarding age, some studies found a decrease^13,24,29,30^with increasing age, while the ESSI general population study found a slight increase^28^. If gender and age differences in social support are found in clinical studies, it remains unclear to what extent these differences are due to the disease or whether the differences already exist in the general population. For a better interpretation of gender and age differences in such clinical samples, it is therefore important to have reliable information about their existence in the general population.

Social support is not only associated with sociodemographic variables but also with behavioral factors. Cigarette smoking and alcohol drinking are such behavioral factors with a high public health impact. Social support is relevant to these behavioral and potentially addictive factors in two ways: (1) explaining the behavior, and (2) in helping people stop drinking and smoking^31–34^.

Social support is also associated with various facets of quality of life, both in clinical samples and in the general population. In our study, we intend to examine the extent to which social support (as measured with ESSI) is related to physical health and mental health, and to what extent the measurement of social support with the 5-item ESSI can be replaced with the short 1-item subscale on social functioning as used in the SF-8.

Temporal stability and long-term changes of social support are insufficiently investigated. Some research has investigated test-retest correlations of social support scales with a short time frame of some weeks, e.g^27,35,36^. , . While there are several large-scale general population studies on long-term changes in physical and mental health, analyses of long-term changes of social support are almost nonexistent in the literature. Such long-term changes are relevant from two perspectives. First, it is a question of which population groups on average experience an increase or decrease in the level of social support over a longer period of time. In addition to such pre-post mean comparisons, it is also important to explore the extent to which individual values of social support change. Even if mean levels remain constant in a population, sharp individual increases and decreases can cancel each other out. Test-retest correlations can be used to estimate how stable individual levels of social support are over time. Findings on this issue are not yet available in the literature, neither with the ESSI nor with other instruments for measuring social support.

The aims of this investigation were (a) to test psychometric properties of the ESSI, based on a large general population study (*n* = 9,681), (b) to test the associations between sociodemographic and behavioral variables and social support, (c) to analyze the association between social support and several variables of health and quality of life, and (d) to test changes and the temporal stability of social support during a six-year period. The study is exploratory in nature and does not contain specific hypotheses.

## **Methods**

### **Sample**

Between 2011 and 2014, the population-based LIFE-Adult Study was conducted, examining a German community sample with 10,000 representatively selected persons^37^ from Leipzig, a city in Eastern Germany with around 550,000 inhabitants at the time of the baseline survey.

The age range was from 18 to 80 years, with a focus on the 40–80 age range according to the study protocol. The main aim of this study was to investigate causes for the development of civilization diseases. Letters of invitation were sent out by mail, and the response rate of this LIFE-Adult Study was 33%. At the study center, the participants underwent several assessment batteries, including collection of sociodemographic variables, medical history, lifestyle factors, and multiple medical examinations. The ESSI and several other questionnaires were also included in this study. Pregnancy and insufficient command of the German language were exclusion criteria. Pregnancy was chosen as an exclusion criterion because the medical examinations might be too laborious for pregnant women.

A follow-up study was performed between 2017 and 2021. All participants of the t1 baseline assessment were invited to answer questions concerning the state of their health and to complete several questionnaires. The baseline (t1) and the follow-up (t2) study were approved by the Ethics Committee of the Medical Faculty of the University of Leipzig, and written informed consent was obtained from all participants. All research was performed in accordance with relevant guidelines and regulations. Further details of the study design have been published elsewhere^37^.

### Instruments

The ENRICHD Social Support Instrument (ESSI)^23,38^was originally developed for patients suffering from myocardial infarction. It focuses on social support by close friends or family. In our study, we used the German version of the ESSI^28^. The questionnaire comprises five items to be rated on a scale of 1 (“never”) to 5 (“always”). One example item is the question, “If you need to talk, is there someone who listens to you properly?”. The ESSI sum score is the sum of the five item scores, resulting in a scale range of 5 to 25. The ENRICHD investigators^38^defined a low level of social support when the ESSI total score was ≤ 18, and when at least two of the five items received a rating ≤ 3.

In addition to the ESSI, several other questionnaires were used in this examination:

The Life Orientation Test-Revised LOT-R^39^is a 10-item instrument for measuring dispositional optimism with the two subscales optimism and pessimism. The Satisfaction With Life Scale^40^comprises five items for assessing general life satisfaction. The Generalized Anxiety Disorder Screener GAD-7^41^is a 7-item questionnaire for measuring anxiety, based on the DSM-IV criteria for generalized anxiety disorders. The Patient Health Questionnaire PHQ-15^42^was developed for measuring physical complaints, based on 15 frequently observed complaints. Finally, the Short Form Health Survey–8 SF-8^43^ measures quality of life with eight single-items scales, from which two subscales can be derived: physical component summary (PCS) and mental component summary (MCS).

Alcohol consumption was assessed with regard to frequency and amount of different alcoholic beverages consumed within the last year, and tobacco use was assessed with questions about former and current smoking and amounts of different tobacco products used. From this data, we derived two categories regarding daily alcohol consumption (< 20 g/day vs. ≥ 20 g/day) and two categories regarding smoking (current smoker no / yes).

Sociodemographic factors were obtained in a structured interview.

#### **Statistical analysis**

Age and gender differences in social support (ESSI) were tested with two-factorial analyses of variance (ANOVAs), using the factors gender (two categories) and age group (five age categories according to Table 1). Internal consistency was calculated using Cronbach’s α coefficient. Effect sizes were calculated using Cohen’s *d*, relating the mean score differences to the pooled standard deviation. Associations between the ESSI scores and other scales were expressed in terms of Pearson correlations. The effects of socioeconomic and behavioral factors on the ESSI score were tested with three-factorial ANOVAs. In addition to the factor of interest, the factors gender and age group were included in these ANOVAs to control for these possible confounders.

**Table 1:** Sociodemographic characteristics of the sample.

	Men (*n* = 4,651)		Women (*n* = 5,030)		Total (*n* = 9,681)	
	M	(%)	M	(%)	M	(%)
Age						
Mean (SD)	57.2 (12.6)		56.2 (12.2)		56.7 (12.4)	
Age group						
≤ 39 years	256	(5.5)	254	(5.0)	510	(5.3)
40–49 years	1,212	(26.1)	1,424	(28.3)	2,636	(27.2)
50–59 years	1,030	(22.1)	1,206	(24.0)	2,236	(23.1)
60–69 years	1,181	(25.4)	1,281	(25.5)	2,462	(25.4)
≥ 70 years	972	(20.9)	865	(17.2)	1,837	(19.0)
Marital status ^(a)^						
Married, living together	2,985	(64.3)	2,809	(55.9)	5,794	(59.9)
Married, living separately	103	(2.2)	130	(2.6)	233	(2.4)
Never married	916	(19.7)	827	(16.5)	1,743	(18.0)
Divorced	527	(11.3)	793	(15.8)	1,320	(13.7)
Widowed	113	(2.4)	465	(9.3)	578	(6.0)
Education ^(a)^						
< 10 years	366	(8.0)	390	(7.8)	756	(7.9)
10–11 years	2,508	(54.8)	2,987	(60.1)	5,495	(57.6)
≥ 12 years	1,703	(37.2)	1,594	(32.1)	3,297	(34.5)
Occupational status ^(a)^						
Working full time	2,319	(50.3)	1,956	(39.3)	4,275	(44.6)
Working part-time	169	(3.7)	743	(14.9)	912	(9.5)
Unemployed	307	(6.7)	294	(5.9)	601	(6.3)
Retired	1,748	(37.9)	1,831	(36.8)	3,579	(37.3)
Other	68	(1.5)	149	(3.0)	217	(2.3)
Income ^(a)^						
< 1000 €	911	(20.6)	1,789	(37.3)	2,700	(29.3)
1000 - < 2000 €	2,282	(51.6)	2,230	(46.5)	4,512	(49.0)
≥ 2000 €	1,228	(27.8)	772	(16.1)	2,000	(21.7)
Socioeconomic level ^(a)^						
Low	893	(19.2)	1,024	(20.4)	1,917	(19.9)
Medium	2,710	(58.4)	3,092	(61.6)	5,802	(60.1)
High	1,036	(22.3)	901	(18.0)	1,937	(20.1)
Smoking ^(a)^						
Current nonsmoker	3,474	(76.3)	3,907	(79.7)	7,381	(78.0)
Current smoker	1,081	(23.7)	996	(20.3)	2,077	(22.0)
Alcohol consumption ^(a)^						
< 20 g/day	2,886	(66.5)	4,354	(92.6)	7,240	(80.1)
≥ 20 g/day	1,451	(33.5)	350	(7.4)	1,801	(19.9)
BMI ^(a)^						
< 25 kg/m²	1,303	(28.2)	2,043	(40.9)	3,346	(34.8)
25 - <30 kg/m²	2,173	(47.0)	1,716	(34.3)	3,889	(40.4)
> 30 kg/m²	1,146	(24.8)	1,242	(24.8)	2,388	(24.8)
^(a)^ missing data not reported						

Socioeconomic status was defined in accordance with the Robert-Koch-Institute’s Gesundheit in Deutschland Aktuell (GEDA) examination^44^, which integrates education, income, and professional position into one index. Income was defined as household net income, divided by the weighted number of household members.

The factorial structure was tested with a confirmatory factor analysis (CFA). As criteria for measuring model fit, we used the comparative fit index (CFI), the Tucker-Lewis index (TLI), and the root mean square error of approximation (RMSEA). The χ² index is not considered an absolute fit indicator because of the large sample size. Measurement invariance across gender and age groups was tested with three models: Model 1 (configural invariance) tests whether the model structure of the unconstrained model is preserved when group membership is taken into account (gender or age groups). Model 2 (metric or weak invariance) is used to additionally check the equivalence of the factor loadings between the groups. Finally, Model 3 (scalar or strong invariance) is used to check the equivalence of the intercepts in addition to the restrictions of Model 2. The statistical calculations were performed with IBM SPSS Statistics (Version 27) and AMOS (Version 5).

## Results

### Sample characteristics

Table 1 presents characteristics of the sample at t1 (*n* = 9,681). Of these participants, 4,987 could be reached at t2 (51.5% of the t1 participants). The mean age of the t1 sample was 56.7 years (range: 18–80), and 52.7% of the sample were women. According to the study protocol, the age range 18–39 years was included but underrepresented.

### Age and gender differences in social support

The ESSI total mean score of the whole sample was M = 22.06 (SD = 3.72). Table 2 presents the ESSI mean scores of the gender and age groups. In total, women reported slightly higher mean levels of social support than men did (*d* = 0.08). There was only a small age effect. With one exception, all group mean values were between 21.46 and 22.20 (Table 2). The highest levels of social support were reported by young women in the age range 18–39 years, but the sample size of this subgroup was relatively low (*n* = 254). The results of the two-way ANOVA for testing the gender and age effects on the ESSI score were: gender (F = 20.4; *p* < 0.001), age group (F = 4.0, *p* = 0.003), and interaction gender * age group (F = 6.1; *p* < 0.001).

**Table 2:** ESSI mean scores for men and women (*n* = 9,681).

Age	Men		Women		d	*p*
	M	(SD)	M	(SD)		
≤ 39 y.	21.89	(3.46)	22.96	(2.53)	0.36	< 0.001
40–49 y.	21.81	(3.76)	22.20	(3.62)	0.11	0.007
50–59 y.	21.46	(4.18)	22.19	(3.61)	0.19	< 0.001
60–69 y.	22.15	(3.80)	22.11	(3.72)	−0.01	0.794
≥ 70 y.	22.20	(3.58)	22.09	(3.72)	−0.03	0.531
All	21.90	(3.82)	22.19	(3.62)	0.08	< 0.001

Using the criterion for low levels of social support as defined by the original ENRICHD investigators, low levels of social support were found for 1,257 participants (13.0% of the total sample). The percentage of low social support was significantly higher for men (14.5%) than it was for women (11.6%) (χ² = 17.49, *df* = 1; *p* < 0.001).

## Psychometric properties of the ESSI

The correlations among the items were between 0.60 and 0.80. Cronbach’s α was 0.91, and the five part-whole-corrected item-total correlations were in the range between 0.73 and 0.84.

Regarding the distribution of the ESSI, there was a skewness (−1.71) in terms of a ceiling effect, which is also reflected in the high mean score (M = 22.1) on the scale with a range from 5 to 25. The maximum score of 25 points was reached by 36.9% of the respondents, and the frequencies of the following scores were 12.5% (24 points) and 9.5% (23 points).

The CFA confirmed the one-dimensional structure of the scale (Table 3). Measurement invariance was tested across gender and age groups. Model 1 assumed the same item-factor assignments across the groups, Model 2 assumed these item-factor assignments and equivalent factor loadings across groups, and Model 3 added equivalent intercepts to the specifications of Model 2. Taking the criteria together, Model 3 (equivalence in factor loadings and in intercepts) fitted the data best.

**Table 3:** Fit indices for multigroup CFA.

Model	χ²	df	χ²/df	CFI	TLI	RMSEA
Unconstrained model	1,368.6	5	273.7	0.991	0.982	0.168
Gender						
Model 1: configural invariance	1,038.6	24	43.3	0.994	0.995	0.093
Model 2: metric (weak) invariance	1,169.2	28	41.8	0.993	0.995	0.092
Model 3: scalar (strong) invariance	1,271.9	33	38.5	0.993	0.996	0.088
Age groups						
Model 1: configural invariance	767.0	62	12.4	0.996	0.997	0.068
Model 2: metric (weak) invariance	991.6	74	13.4	0.994	0.997	0.071
Model 3: scalar (strong) invariance	1,095.3	89	12.3	0.994	0.997	0.068

## Associations between sociodemographic variables, behavioral variables, and social support

The relationship between multiple sociodemographic variables and social support is illustrated in Table 4. High education, high income, high socioeconomic level, being married (and living together), and being employed resulted in high levels of social support. Tobacco smokers and alcohol drinkers reported less support than nonsmokers and non-drinkers. Though adipose people reported slightly lower levels of social support than the other groups, BMI had was not statistically significantly associated with social support.

**Table 4:** ESSI mean scores, broken down by sociodemographic and behavioral variables.

	Men (*n* = 4,651)		Women (*n* = 5,030)		Total (*n* = 9,681)	
	M	(SD)	M	(SD)	M	(SD)
Marital status (F = 172.1, *p* < 0.001)						
Married, living together	22.7	(3.1)	22.9	(3.0)	22.8	(3.1)
Married, living separately	20.0	(5.0)	21.3	(4.3)	20.7	(4.7)
Never married	20.6	(4.3)	21.6	(4.0)	21.1	(4.2)
Divorced	20.4	(4.8)	21.0	(4.3)	20.7	(4.5)
Widowed	20.9	(4.2)	21.5	(3.9)	21.4	(4.0)
Education (F = 17.1; *p* < 0.001)						
≤ 10 years	21.4	(4.2)	21.7	(4.1)	21.6	(4.1)
10–11 years	21.8	(4.0)	22.1	(3.6)	22.0	(3.8)
≥ 12 years	22.3	(3.4)	22.5	(3.4)	22.4	(3.4)
Occupation (F = 3.5, *p =* 0.015)						
Working full time	22.1	(3.5)	22.4	(3.4)	22.3	(3.4)
Working part-time	21.2	(4.1)	22.4	(3.3)	22.2	(3.5)
Unemployed	19.6	(5.1)	20.3	(4.9)	20.0	(5.0)
Retired	22.1	(3.8)	22.1	(3.7)	22.1	(3.7)
Other	22.2	(3.7)	22.5	(3.2)	22.4	(3.4)
Income (F = 41.5, *p* < 0.001)						
< 1000 €	20.8	(4.7)	21.8	(4.0)	21.5	(4.3)
1000 - < 2000 €	22.0	(3.6)	22.3	(3.4)	22.2	(3.5)
≥ 2000 €	22.4	(3.4)	22.7	(3.1)	22.5	(3.3)
SES (F = 126.8, *p* < 0.001)						
Low	20.3	(4.8)	20.9	(4.5)	20.6	(4.6)
Medium	22.1	(3.6)	22.4	(3.4)	22.3	(3.5)
High	22.7	(3.1)	23.0	(2.8)	22.8	(3.0)
Tobacco (F = 53.7, *p* < 0.001)						
Current nonsmoker	22.2	(3.6)	22.4	(3.5)	22.3	(3.5)
Current smoker	21.1	(4.3)	21.6	(4.0)	21.3	(4.2)
Alcohol (F = 10.0, *p =* 0.002)						
< 20 g/day	22.0	(3.8)	22.3	(3.5)	22.2	(3.6)
≥ 20 g/day	21.8	(3.8)	21.8	(3.9)	21.8	(3.8)
BMI (F = 1.5, *p* = 0.226)						
< 25 kg/m²	21.7	(3.9)	22.4	(3.4)	22.1	(3.6)
25 - <30 kg/m²	22.2	(3.5)	22.1	(3.6)	22.2	(3.6)
≥ 30 kg/m²	21.6	(4.2)	21.9	(4.0)	21.8	(4.1)

## Relationship between social support, quality of life, and health-related variables

Table 5 presents the correlations between social support and several other variables. The strongest association was found for life satisfaction (*r* = 0.43). Among the eight dimensions of the quality of life questionnaire SF-8, the correlations were highest for social functioning (*r* = 0.29), immediately followed by mental health (*r* = 0.28). The correlation between the mental component summary score (*r* = 0.31) was even slightly higher than that of social functioning.

**Table 5:** Correlations between ESSI and other variables.

Variable	*r*
LOT-R Optimism	0.28 ***
LOT-R Pessimism	−21 ***
SWLS: Life satisfaction	0.43 ***
GAD-7: Anxiety	− 0.27 ***
PHQ-15: Complaints	− 0.21 ***
SF-8 Physical functioning	0.17 ***
SF-8 Role Physical	0.18 ***
SF-8 Bodily pain	0.12 ***
SF-8 General health	0.22 ***
SF-8 Vitality	0.25 ***
SF-8 Social functioning	0.29 ***
SF-8 Role emotional	0.26 ***
SF-8 Mental health	0.28 ***
SF8 – Physical Component Summary	0.15 ***
SF8 – Mental Component Summary	0.31 ***

***: *p* < 0.001.

## Changes in social support over six years

Of the 9,681 people included in the t1 assessment, 4,987 could be reached at t2 (51.5% of the t1 participants). To fairly compare the t2 scores with the t1 scores, we limited our analysis here to those people who attended both examinations, t1 and t2 (*n* = 4,987). First of all, the total mean score of the t1 examination was 22.33 for the 4,987 t2 participants. This score is higher than the mean of the original 9,681 persons (M = 22.06). Those who participated at t1 but not at t2 (drop out, *n* = 4,694) showed a t1 mean score of only M = 21.77 ± 3.99, which results in an effect size of *d* = 0.15 (*p* < 0.001) for the comparison between the t2 participants and the t2 non-participants.

Table 6 presents mean scores and test-retest correlations for age and gender groups. The comparison of the t1 and the t2 scores shows that both total mean scores were nearly equal, the effect size was only *d* = 0.01. There was only one subgroup which showed a remarkable trend: Women of the oldest age group reported a reduction of social support with an effect size of *d* = 0.17. All other group effect sizes were below 0.10.

**Table 6:** The comparison of the t1 and t2 scores of social support, restricted to t1 and t2 participants (*n* = 4,987).

	t1		t2		d(t2,t1)	*p*	*r*_tt_
	M	(SD)	M	(SD)			
Men 18–39 y.	21.99	(3.07)	21.96	(3.65)	−0.01	0.937	0.55
Men 40–49 y.	22.17	(3.47)	22.29	(3.37)	0.04	0.372	0.59
Men 50–59 y.	21.64	(4.01)	21.95	(4.05)	0.09	0.051	0.62
Men 60–69 y.	22.47	(3.45)	22.45	(3.48)	−0.01	0.899	0.61
Men 70–80 y.	22.47	(3.18)	22.43	(3.46)	−0.01	0.784	0.52
Men total	22.19	(3.52)	22.27	(3.59)	0.02	0.234	0.59
Women 18–39 y.	23.14	(2.37)	22.86	(3.20)	−0.09	0.320	0.40
Women 40–49 y.	22.44	(3.38)	22.43	(3.42)	0.00	0.971	0.59
Women 50–59 y.	22.38	(3.46)	22.29	(3.74)	−0.03	0.431	0.68
Women 60–69 y.	22.42	(3.43)	22.31	(3.49)	−0.04	0.341	0.64
Women 70–80 y.	22.39	(3.30)	21.86	(3.86)	−0.17	< 0.001	0.62
Women total	22.45	(3.36)	22.29	(3.59)	−0.05	0.007	0.62
Total	22.33	(3.44)	22.28	(3.59)	−0.01	0.284	0.61

*d*(t2,t1): effect size of the difference between the t1 and t2 scores; *r*_tt_ : correlations between the t1 and t2 scores.

Table 6 also presents the correlations between the t1 and t2 measurements. Considering the total sample, the correlation coefficient was 0.61. The group with the highest temporal stability (*r* = 0.68) were women between 50 and 59 years of age, and the lowest stability scores were observed for the youngest age group, especially the young women.

## Discussion

The first research question concerned the psychometric quality of the ESSI. The reliability (Cronbach’s α = 0.91) was very good. This is in line with coefficients obtained from other studies: α = 0.93^29^in a sample of the general population; α = 0.89^13^in healthcare workers; α = 0.88^15^in cell transplantation survivors, and α = 0.88^27^in cardiac patients. The CFA also confirmed the one-factorial structure and measurement invariance across gender and age groups. This means that gender and age comparisons are fair when using the ESSI. One disadvantage of the scale, however, is its skewness, with a concentration on high levels of social support, which has also been observed in previous studies of the general population^29^and patients with cardiovascular diseases^28^. The clear one-dimensional structure of the ESSI has the psychometric advantage that the sum score can be used without restrictions. The associated disadvantage is that it is not possible to record several different components of social support.

Gender differences in social support proved to be small in magnitude. Women reported higher levels of social support than men (*d* = 0.08). The highest gender difference was observed in the youngest group (18–39 years), however, the sample size was lowest in that age group, which limits the generalizability of this finding. Concerning the age groups of 60 years and above, gender differences disappeared nearly totally.

Using mean scores of the Oslo Social Support Scale OSSS-3 in another large general population sample, the gender differences were also low, with an effect size of *d*= 0.03^24^. Based on the Lubben social support scale and a criterion that defined a low level of social support (called “social isolation”), a general population study also found a higher frequency of low social support in men (13.8%) in comparison to women (10.9%)^30^. These proportions are similar to our results (14.5% and 1.6%).

When interpreting the slightly higher values of social support for women compared to men, various aspects can be taken into account. Social support is related to personality traits. Agreeableness is associated with social support, and this trait is on average more pronounced in women than in men^45^. For men, independence is more important and has a more positive connotation than for women^46^. This may also mean that men may have fewer problems admitting that their social network is small. Related to this is the fact that men are more satisfied with the size of their social network, even if this network is not objectively large^47^. Men may have smaller social networks since their social needs are often supported by their female partners, while women are more active in maintaining social contacts outside the home^48^. In the interpretation it should also be noted that social isolation is not identical with a low level of social support. A single instrument such as the ESSI is not sufficient for an in-depth analysis of gender differences in social support with all its various objective and subjective components.

Age differences were also small in magnitude with no linear trend for either gender. The only peculiarity in the age course was the relatively high level for young women, which, as already mentioned above, may be due to the relatively low sample size. The lack of a clear decrease of social support with increasing age (at least up to the higher age limit of 80 years in this study) means that public health efforts to strengthen social networks should not only consider elderly people. The age and gender differences obtained in our large general population sample help interpret the scores of groups of patients with specific age and gender distributions.

The ESSI total mean score of our sample was 22.06, which is very similar to the mean of a large German sample of cardiac patients (M = 21.8)^28^and slightly higher than the levels of German healthcare workers during the COVID pandemic (M = 20.65)^13^. It is not surprising that among patients, the level of social support is not lower than the level of general population studies since patients may activate their social network for coping with the disease. The similarity of the ESSI mean scores and the similarity of the reliability coefficients (see above) between clinical and non-clinical samples means that the ESSI can be used in a similar way both in clinical trials and in the general population.

It is interesting to consider the relationship between social support and social functioning, a variable often found in quality of life questionnaires. The correlation between social support and social functioning (as measured with the SF-8) was only *r* = 0.29 in our study, and other studies also reported relatively low correlations, e.g., *r*= 0.24 in cardiovascular patients^28^. It is possible for patients to simultaneously experience low levels of social functioning and high levels of social support as these two factors are distinctly different concepts, and social support cannot be replaced with a social functioning scale in epidemiological or clinical studies.

High levels of socioeconomic status, in particular education and income, were associated with high degrees of social support in this study. People with disadvantages regarding income, education, and employment are much more likely to have smaller and less reliable social networks^17,30^. Better chances for good education for all people is therefore a means that may improve social relatedness, which may then contribute to reduce inequalities in social networks and in health^49,50^. The results of our study suggest that efforts to strengthen public health should incorporate providing opportunities to strengthen social networks, especially for those people of low socioeconomic status.

Social support was associated with cigarette smoking and, to a lower degree, with alcohol consumption: cigarette smokers and alcohol drinkers reported lower levels of social support than non-smokers and non-drinkers, which is in line with previous studies^31,51^. These relationships suggest that public health interventions aimed at strengthening social networks may also have positive effects on alcohol and tobacco use. Social support is not only important for explaining behaviors such as alcohol consumption and smoking, but also for quitting drinking and smoking. Individuals who are trying to stop their alcohol and nicotine use benefit greatly from social support in doing so^32–34,52^. It should be noted, however, that the associative relationships between social support and alcohol and tobacco use are not necessarily causal. These relationships may be moderated by socioeconomic status factors, such that individuals with low social status are on average both less likely to experience social support and more likely to engage unhealthy behavior such as addictive nicotine or alcohol consumption.

Social support was correlated with satisfaction with life, optimism, low degrees of anxiety, low levels of physical complaints, and with all eight components of the quality of life questionnaire SF-8. This supports findings from other studies (e.g.^28^), that also found associations with variables of mental health and, to a lower degree, physical health. It is interesting to note that the mental component summary score MCS was more strongly correlated with the ESSI score (*r* = 0.31) than the physical component summary PCS (*r* = 0.15), and even higher than the social functioning scale of the SF-8 (*r* = 0.29). As already discussed above, social support (as measured with the ESSI) and social functioning (as measured with the SF-8) are not identical.

During the 6-year period from the baseline assessment to follow-up, the ESSI mean score remained nearly unchanged (*d*= 0.01), which was to be expected considering the negligible age dependency of social support. Only one group (women, 70–80 years) reported a significant decline in social support. In sum, the changes in social support only partly support the thesis that social networks shrink with age^53^.

Despite the temporal stability of the total mean score, change did occur from an individual perspective; the test-retest correlation was 0.61 for the total group. This coefficient is slightly lower than the 6-year test-retest stability of the amount of bodily complaints when assessed with the PHQ-15 (*r*= 0.66^54^). In the literature, there are several studies for testing the test-retest reliability of social support scales with relatively short time intervals of several weeks^27,35,36^, but it is plausible that these coefficients are not comparable with those of a 6-year interval. The 6-year temporal stability of women (*r* = 0.62) was only slightly higher than that of men (*r* = 0.59) in our study. For men as well as for women, the age groups with the highest stability were those between 50 and 70 years of age. This indicates that social networks tend to be the most stable in the decades immediately before and after entering retirement as younger people experience more variation in their family life and their other social relationships in the course of settling into adulthood, while people over 70 years are prone to experience shifts in their close social circles due to changes like moving and loss.

When comparing the respondents of the follow-up examination with those who participated at t1 but not at t2, the mean ESSI score of the complete responders was significantly higher than that of the dropouts (*d*= 0.15). This means that participants with complete data sets represent a positive selection since t1 participants with lower levels of social support were more likely to be lost over the course of the study period, a phenomenon that has also been observed in other longitudinal studies, e.g.^55,56^, and one that limits the generalizability of our findings concerning temporal changes.

Some *limitations*of this study should be mentioned. There was a certain degree of sampling bias. The participants in the baseline assessment were slightly healthier than those who refused participation^57^. Only about half of the t1 participants could be included in the t2 assessment, and the comparison of the dropouts with the completers showed an additional bias towards healthy participants. Moreover, the response rate was low (33% for the t1 assessment). The sample was not representative of the German general population in terms of age distribution. Regarding gender, the proportion of men in the age rage of 40–80 years is 51.2% acording to the Federal Statistical Office of Germany DESTATIS^58^, which is similar to the 52.7% in ou study. In terms of age, however, the youngest age group was significantly underrepresented. In addition, the sample was a community sample that only included residents of a single city in Germany. These circumstances limit the generalizability of the study. The end of the follow-up study period coincided with the beginning of the COVID-19 pandemic, and this may have influenced the level of social support.

While the ESSI is a suitable instrument for measuring social support in general, it does not allow for considering specific components of social support. In contrast to that, the short Oslo Social Support Scale OSSS-3, which only has three items nevertheless measures separate items for each of those three components: close network, concern and interest of others, and neighbors. This breadth of content of the OSSS-3 is offset by the disadvantage of its lower reliability (α = 0.64^24^). This relatively low reliability can only be attributed to a lesser extent to the fact that the number of items in the OSSS-3 is lower than that of the ESSI, since the mean correlation of the OSSS-3 items was only 0.38^24^, while the correlations of the ESSI items in our study were markedly higher. When interpreting the results of our study, it should be borne in mind that the ESSI measures the functional component of social support and not the quantitative scope of the social network.

From a conceptual point of view, social support can be understood as a resource variable with a direct effect on health-related dimensions, or a variable buffering the effects of stressful events on health outcomes^59^, while yet other researchers consider social support a mediating variable^60,61^. These conceptualizations require different statistical approaches. In our analyses, we restricted ourselves to simple correlations with other variables, without making assumptions on causal relationships. Because of the exploratory nature of the study, no targeted hypotheses were generated and tested either.

In summary, the ESSI proved to be a reliable instrument for effectively assessing social support in a community sample. Lack of social support was particularly prevalent among individuals with low socioeconomic status, those with low physical and especially mental health, and those with higher substance use. This suggests that, from a public health perspective, strengthening social networks may be useful for enhancing public health. The general population results reported here can be used for better interpreting results from clinical studies.

## Data Availability

Data cannot be shared publicly because of data protection reasons. Data availability is only possible via a project agreement. Data are available from the LIFE-Adult Study Institutional Data Access / Ethics Committee (contact via https://ldp.life.uni-leipzig.de/) for researchers who meet the criteria for access to confidential data.
